# Ag(i)-mediated self-assembly of anisotropic rods and plates in the surfactant mixture of CTAB and Pluronics†

**DOI:** 10.1039/c8ra10517k

**Published:** 2019-02-05

**Authors:** Hyon-Min Song, Jeffrey I. Zink

**Affiliations:** Department of Chemistry, Dong-A University Busan 604-714 South Korea hyonmin1@dau.ac.kr +82-51-200-7259 +82-51-200-7257; Department of Chemistry and Biochemistry, University of California Los Angeles California 90095-1569 USA

## Abstract

One-dimensional (1D) metallogels are commonly observed in metal-coordinated complexes, but there are not many examples of soft crystalline solids which are generated by the self-assembly of metal–polymer complexation in a non-gel state. In a continued effort to obtain 1D materials by utilizing the tendency of Pluronic triblock copolymers to be micellized anisotropically at an elevated temperature, we investigate Ag(i)-mediated self-assembly of the surfactant mixture of Pluronic copolymers and cetyltrimethylammonium bromide (CTAB). At sufficiently high temperature, Pluronic copolymers are known to organize into many crystalline mesophases, such as body-centered-cubic, hexagonal, and lamellar phases. Four Pluronics of L-31, L-64, P-123, and F-108 were studied, and at the concentration of 17.9%, macroscale 1D rods with the aspect ratios ranging from 3.07 to 12.8 are obtained. At the concentration of 35.7%, anisotropic two dimensional (2D) planar plates are observed. These planar structures were believed to be generated from 2D lamellar mesophases, which is consistent with the general phase diagram of Pluronic copolymers that shows lamellar phase with the highest concentration. In the absence of ascorbic acid, rods and plates of larger size are produced. Rather than as a reductant, ascorbic acid is thought to play the roles of an agent to increase the hydrophilicity, and as a mediator to determine the dimension of rods and plates by hindering the long range self-assembly of alkyl chains. Dehydration by the addition of AgNO_3_, and the increase of hydrophobicity enable self-assembly of alkyl groups of CTAB and Pluronics and promote the formation of crystalline soft solids.

## Abstract 2

Macroscale anisotropic rods and plates were generated by the self-assembly of CTAB and Pluronics in the presence of AgNO_3_.

## Graphical abstract

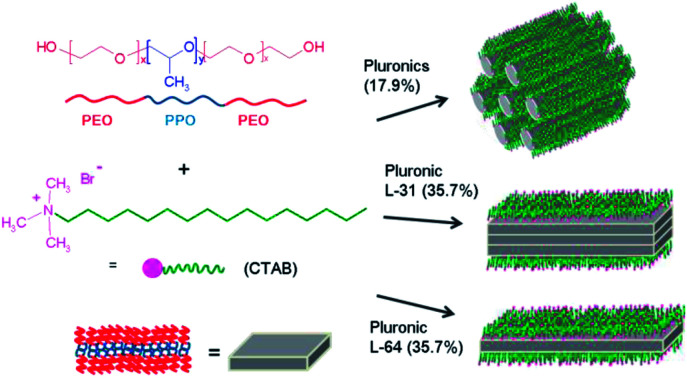

## Introduction

Pluronic triblock copolymers are widely used as a soft template as well as a cell culture medium and drug delivery vehicles for their biocompatibility. They are constructed as PEO (polyethylene oxide)–PPO (polypropylene oxide)–PEO triblocks. Normally they exist as micelles due to the difference of hydrophobicity between PEO and PPO, and accordingly they are prepared as micellar core–shell nanoparticles (NPs) with metallic cores.^1^ These micellar NPs respond to the change of biological conditions such as temperature.^2^ Thus, Pluronic copolymers are termed pharma copolymers with their biological viability. They are used as vehicles for cancer^3^ and gene therapy,^4^ and are applied for stimuli–responsive drug delivery^5^ and controlled release.^6^ For their biocompatibility and amphiphilicity, oral doses have even been tried to compensate for the poor drug solubility in water.^7,8^

A common finding in the study of phase behaviors of Pluronic polymers is that at sufficiently high temperature, they integrate into crystalline mesophases such as cubic, hexagonal, and lamellar structures.^9,10^ When inorganic compounds are added to Pluronic copolymers, they have two effects. One is the reduction of temperature and concentration at which micelles are formed.^11^ The other effect is that anisotropic rod-like micelles are formed at lower temperature. It is known that the increase of hydrophobicity causes PEO coronas to be dehydrated. While these dehydrated PEO coronas are combined with PPO hydrophobic cores, micellar core radius increases, and spherical micelles do not maintain shape stability and change into anisotropic prolate ellipsoids.^12^ Micellization and gelation as well as the transformation of spherical micelles into rod-like micelles are observed in P-85 at 38 °C.^13^ The addition of LiCl is also known to produce lyotropic liquid crystalline hexagonal mesophases.^14^ Anisotropic wormlike micelles are also observed from Pluronic P-84 upon the addition of NaCl.^15^ Whether it is based on molecular dynamic simulation^16^ or on X-ray, neutron, and light scattering experiments,^15,17^ the existence of anisotropic soft solids with crystalline structures have been suggested.

It is reported here that by the addition of AgNO_3_ in the presence of CTAB and Pluronic copolymers, anisotropic soft solids are obtained rather than the usual metallogels in metal–block copolymer complexation. The surfactant mixture of cetyltrimethylammonium bromide (CTAB) and Pluronics are known to undergo self-assembly between the long alkyl chains of CTAB and a large number of methyl groups in the hydrophobic PPO cores, while hydrophilic ammonium head groups in CTAB reside in the interface between hydrated coronas and hydrophobic cores.^18^ Seedless formation of Pd nanorods was reported in the surfactant mixture of Pluronics and CTAB.^19^ In case of AgNO_3_, macroscale rods are obtained as separable soft solids, presumably due to the strong complexation of Ag(i) within Pluronic EO groups. The growth into one dimension is performed with four Pluronics, two existing as liquids (L-31, L-64), one as a paste (P-123), and one as a flake (F-108). Their size is micron scale, for example, with a dimension of 12.2 μm × 1.3 μm (length × width) of the rods prepared from Pluronic L-64. The novelty of this work is that it is one of rare examples of mesoscale self-assembly of metal–block copolymers into soft crystalline solids, and the surfactant mixture of CTAB and Pluronics as soft templates described in this study can be extended to possible anisotropic self-assembly of other transition metal–block copolymers. In fact, Nalbandian and coauthors in early 1970s reported the effect of F-127/AgNO_3_ gels on the repair and growth of the burned skins as well as the antibacterial effect against *Pseudomonas* infection.^20^ CTAB is also used in this study as the surfactant mixture. There is an example that CTAB assists self-assembly of organic heterocycles into optically active one-dimensional (1D) nanofibers by inducing the formation of cylindrical micelles.^21^ This study is another example that the mixture of CTAB and soft matter are organized into distinctive anisotropic soft materials.

## Experimental section

All chemicals including Pluronic triblock copolymers (CAS 9003-11-6) with the number average molecular weights (*M*_n_s) of ∼1100 (product name Pluronic L-31), ∼2900 (Pluronic L-64), ∼5800 (Pluronic P-123), and ∼14 600 (Pluronic F-108) were purchased from Sigma-Aldrich. Aqueous CTAB solution (0.2 M) was recrystallized twice. X-ray diffraction (XRD) patterns were obtained with Rigaku Ultima IV diffractometer using CuKα radiation (*λ* = 1.54056 Å) in θ–θ mode. JAC ultrasonic 1505 (150 W, 40 kHz) was used for sonication. Transmission electron microscopy (TEM) images were obtained with JEOL JEM 2010FX operating at 200 kV. Scanning electron microscopy (SEM) images were taken with JEOL JSM 6700F with an operating voltage of 5.0 kV. Fourier transform infrared (FTIR) spectra were recorded with JASCO FTIR-4600 type A, ranging from 650 cm^−1^ to 4000 cm^−1^ with a resolution of 4 cm^−1^ and with an average scan of 8. Without adding KBr or making KBr pellets, sample was placed on the single-reflection ATR accessory and the spectra were measured while the pressure applicator was implemented.

### Preparation of macroscale 1D rods

Aqueous solution of 17.9% and 35.7% Pluronic copolymers were used in this study. For the preparation of 17.9% aqueous solution, 2.5 g of Pluronic block copolymers was dissolved in 11.5 mL of H_2_O. For preparing 35.7% aqueous solution, 5.0 g of Pluronic copolymers was dissolved in 9.0 mL of H_2_O. For homogenization, the aqueous solution of Pluronic copolymers was repeatedly vortex-mixed (×10) and sonicated for 15 min at room temperature, and it was placed on bench for at least 24 h before being used for the preparation of macroscale rods and plates. For preparing 1D rods, Pluronic copolymer (3.0 mL, 17.9%) and CTAB (2.5 mL, 0.2 M) were mixed, and AgNO_3_ (0.5 mL, 0.015 M) was added to this mixture. This reaction mixture was vortex-mixed for 15 s, and sonicated at 23 °C for 10 min. Ascorbic acid (0.65 mL, 0.568 M) was added, and the mixture was vortex-mixed for 15 s, followed by the sonication at 43 °C for 25 min. The mixture was placed in the heating oven for 1 h at 70 °C without stirring or agitation. White precipitates were observed and they were separated by centrifuging at 2500 rpm for 3 min. Purification was repeated (×2) by centrifugation and washing with 5.0 mL of H_2_O. For the preparation of two-dimensional plates, other conditions are the same except the concentration of Pluronic copolymers (35.7%, L-31 or L-64).

## Results and discussion

The preparation of macroscale rods from the surfactant mixture of CTAB and Pluronics is one step method without using any seeds. During homogenization after the addition of AgNO_3_ to the surfactant mixture of Pluronics and CTAB, thick clouds were observed. When it is heated to 70 °C and before cooling to room temperature, white precipitates are settled. The gelation was not observed. The concentrations of 17.9% aqueous solution of L-31, L-64, P-123, and F-108 are 0.163 M, 62.1 mM, 30.8 mM, and 12.2 mM, respectively. These are sufficiently higher than the critical micelle concentrations (CMCs) of L-31 (0.02 M),^22^ L-64 (8.8 mM),^22^ P-123 (0.0044 mM),^23^ and F-108 (0.022 mM),^23^ so that micelles are formed with hydrophobic PPO cores which are surrounded by hydrophilic PEO regions. As the concentration of Pluronic copolymers increases, the temperature over which micellization occurs decreases so that micelles are formed at lower temperature. For example, the temperature of micellization of Pluronic F-108 (0.017 mM) is 40.5 °C, while Pluronic F-108 (6.85 mM) micellizes at 21.0 °C.^24^ All Pluronic copolymers in this study micellize at lower temperature than the sonication temperature (43 °C). The concentrations in fact, are much higher than CMCs, thus they are able to self-assemble into micelles before they are heated.

Typically, CMC decreases as temperature increases due to the increased hydrophobicity.^10^ It is known that the reversible dehydration of PPO blocks is closely related with micelle formation, and PPO blocks with at least 10 PO groups are required to obtain micelles.^10^ Further increase of temperature produces cubic crystalline mesophases, and it is also known that the gel state of Pluronics is the evidence of long range order of cubic mesophases.^25^ At a specific temperature, isotropic micelles integrate into body-centered cubic, hexagonal, and lamellar mesophases in sequence with increasing concentration.^10^ The other aspect of phase behavior of Pluronic copolymers is the effect of the added salt, in particular metal nitrate. By the addition of transition metal nitrate, Pluronic copolymers change into hexagonal, cubic and even tetragonal mesophases.^26^ Zinc nitrate causes the transformation of liquid crystalline phase into solidified mesostructures.^27^ The addition of silver nitrate is critical to obtain rigid rods and plates in this study. The simple mixture of two surfactants, CTAB and Pluronic copolymers hardly produces rigid materials. Silver nitrate is thought to reside in PEO coronas, and to disrupt hydrogen bonding between water and EO groups. Dehydration of PEO coronas occurs and due to the increase of hydrophobicity, anisotropic rigid rods and plates are believed to be generated.

**Fig. 1:**
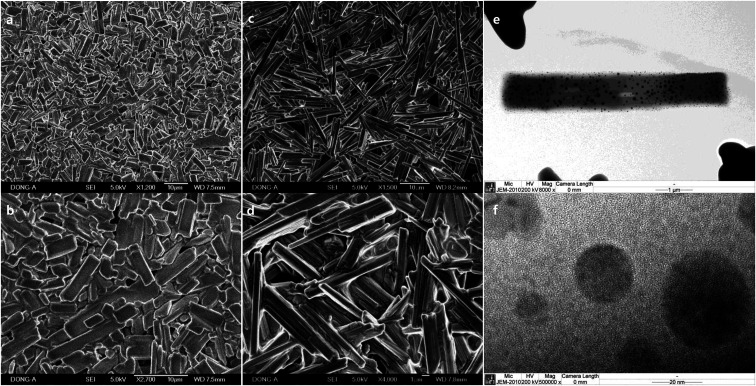
SEM images of different magnification of macroscale rods prepared with (a) and (b) Pluronic L-31 (17.9%), and (c) and (d) Pluronic L-64 (17.9%). TEM images of (e) the rod obtained with Pluronic L-64 (17.9%) and (f) Ag NPs uncovered during the exposure to the electron beam.

**Fig. 2:**
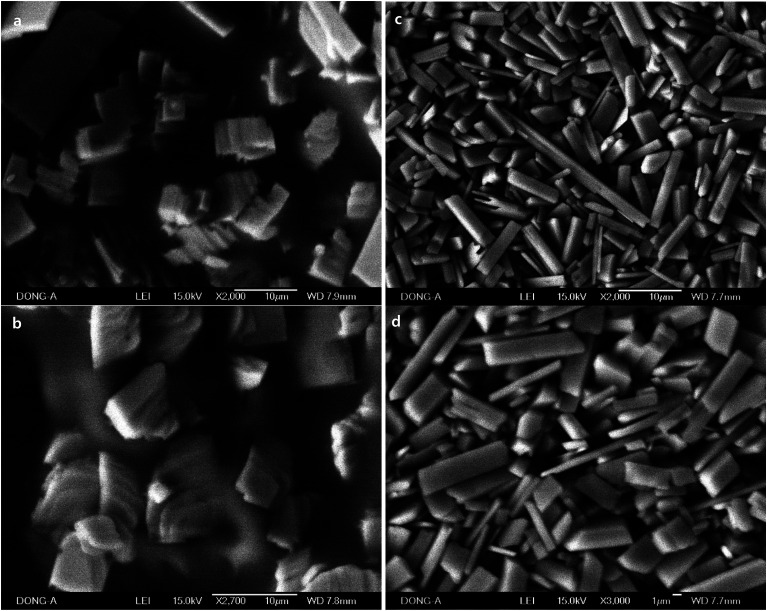
SEM images of different magnification of macroscale rods and plates prepared with (a) and (b) Pluronic L-31 (35.7%), and (c) and (d) Pluronic L-64 (35.7%).

Macroscale rods which were prepared with Pluronic L-31 (17.9%, Fig. 1a and b), and Pluronic L-64 (17.9%, Fig. 1c and d) were observed in SEM images. In TEM images (Fig. 1e and f), Ag NPs are uncovered on the rods obtained with Pluronic L-64 (17.9%) during the exposure to the electron beam. Severe strains exist in those Ag NPs. As the size increases, the strain follows to minimize surface energies, particularly in noble metal NPs with face-centered-cubic (fcc) symmetry. Twin boundaries normally accompany in those nanosize noble metals. Similar uncovering of Ag NPs upon the exposure to the electron beam is studied in the self-assembly of silver carboxylates.^28^ When the concentration of Pluronic copolymers is 35.7%, the aspect ratio decreases significantly to 2.05 (±1.11) (L-31, Fig. 2a and b) and to 3.06 (±1.42) (L-64, Fig. 2c and d). In case of Pluronic L-31, the stacking of plates was observed throughout the samples, while in Pluronic L-64 the anisotropic plates without stacking were observed.

**Fig. 3:**
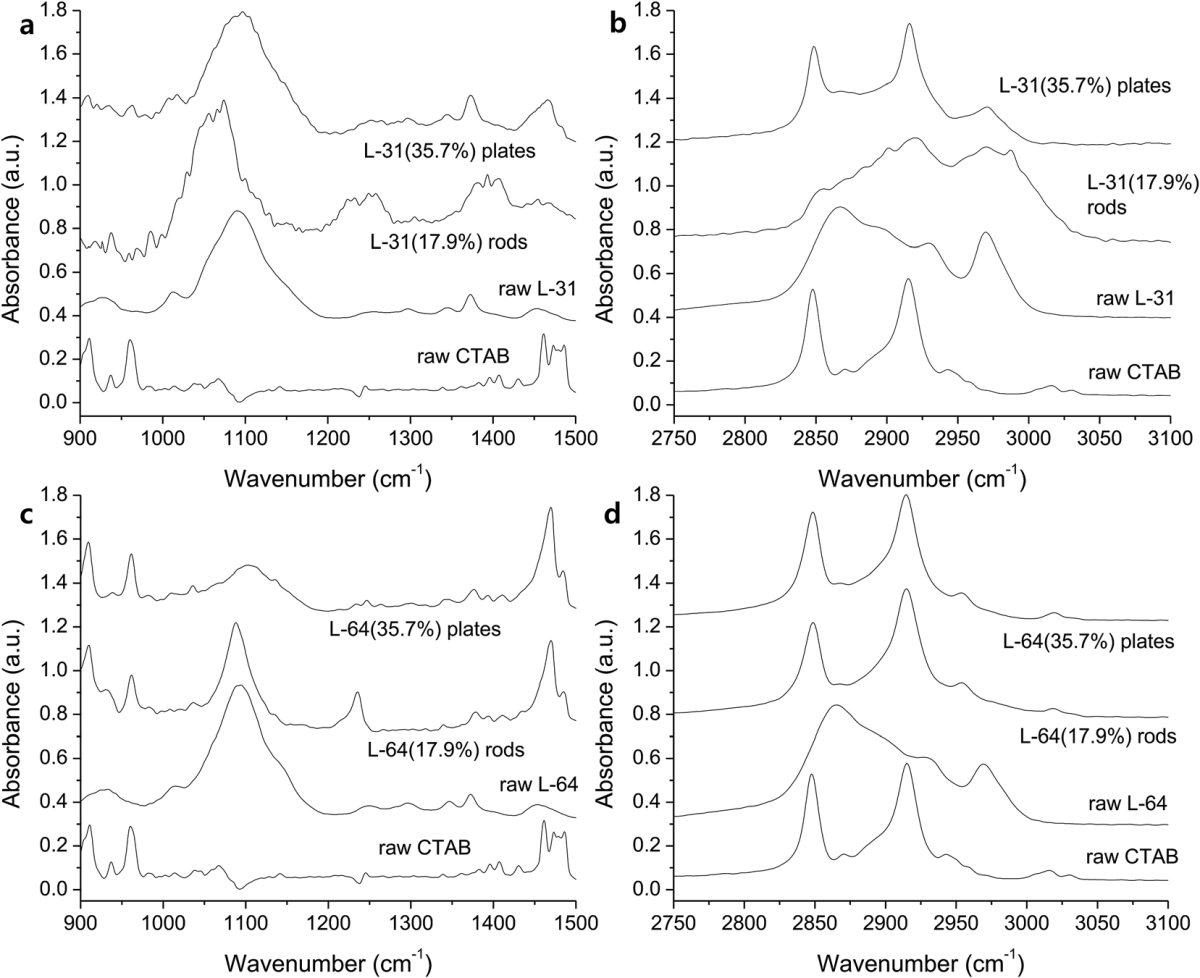
IR spectra of the rods prepared with Pluronic L-31 in the range of (a) 900–1500 cm^−1^ and (b) 2750–3100 cm^−1^, and with Pluronic L-64 in the range of (c) 900–1500 cm^−1^ and (d) 2750–3100 cm^−1^.

The bonding characteristics within EO and PO regions and the degree of self-assembly are investigated with FTIR. An increase of Pluronic concentration from 17.9% to 35.7% produced C–O stretching band close to that of the raw L-31 (Fig. 3a), which implies that more hydrophobic environment causes the removal of water around the micelles and this dehydration in 35.7% of Pluronics causes the peaks near to those of the pure L-31 (Fig. 3a).^29^ The red-shift of C–O stretching in L-31 (17.9%), which is centered at 1063 cm^−1^, is presumably due to the interaction of Ag(i) with oxygen in the hydrophilic environment and the weaker C–O bonding. The shift of C–O stretching band is an important indicator of the degree of hydrophilicity in the solution. In general, hydrophilic interaction of ether groups with H_2_O through hydrogen bonding makes C–O stretching red-shifted. The more hydrophobic Pluronic copolymer is, the more PO groups are dehydrated and in the less polar environment PO groups are placed, which makes the interaction of ether oxygen with H_2_O inefficient and thus C–O stretching is blue-shifted.^30^ The peaks at 1226 and 1253 cm^−1^ in Pluronic L-31 (17.9%) are CH_2_ wag vibrations (Fig. 3a),^31^ and two separate peaks imply two different environments CH_2_ groups are placed, one in the hydrophilic regions at 1253 cm^−1^ due to the increased interaction with H_2_O and the other in the hydrophobic region at 1226 cm^−1^.^32^ With regard to the effect of CTAB on the self-assembly, alkyl chains from CTAB are not pronounced between 900 – 1500 cm^−1^ (Fig. 3a). The structure of L-31 is EO_2_PO_16_EO_2_. Relatively small number of PO chains does not make efficient self-assembly with CTAB alkyls. The small influence of CTAB on the self-assembly of Pluronic L-31 is reflected in alkyl stretching vibrations (Fig. 3b). In the hydrophobic environment (35.7%), dehydrated state of the solution makes CH_2_ symmetric stretching (2848 cm^−1^) and antisymmetric stretching (2916 cm^−1^) more pronounced, which makes the spectra similar to CTAB (Fig. 3b).^33^ However, CH_3_ stretching vibration (35.7%), which is derived from methyl groups in PPO blocks, is also present at 2980 cm^−1^ (Fig. 3b). This implies that CTAB does not govern the spectra, presumably due to the inefficient self-assembly of CTAB alkyl chains with a short chain of PPO in Pluronic L-31.

When rods are grown in more hydrophobic solution, relatively weak and broad C–O stretching band is observed (L-64, 35.7%, Fig. 3c). The broad peak at 1110 cm^−1^ with a full width half maximum (FWHM) of 67.7 cm^−1^ is similar to the peak of raw L-64 at 1093 cm^−1^ (FWHM of 62.0 cm^−1^). CH_3_ stretching mode at 2970 cm^−1^ (Fig. 3d), which is significant in the raw Pluronic L-64 becomes weakened in rods and plates, while two peaks from CH_2_ symmetrical stretching at 2848 cm^−1^ and CH_2_ antisymmetrical stretching at 2916 cm^−1^, which are derived from CTAB, are still dominant (Fig. 3d). The shift of these peaks is not observed, which means that self-assembly in the dehydrated alkyls between CTAB and Pluronics is driven by the hydrophobic interaction during the segregation of water from methyl groups of Pluronics, and self-assembly is not affected by any electrostatic interactions in aqueous medium.^30^ The C–O stretching band at 1090 cm^−1^ becomes sharper in more hydrophilic environment (17.9%, FWHM of 41.0 cm^−1^, Fig. 3c). Similar to the rods obtained with Pluronic L-31 (17.9%), slight red-shift of C–O stretching vibration at around 1100 cm^−1^ occurs and it is due to the stronger complexation of Ag(i) within hydrophilic regions (Fig. 3c).

**Fig. 4:**
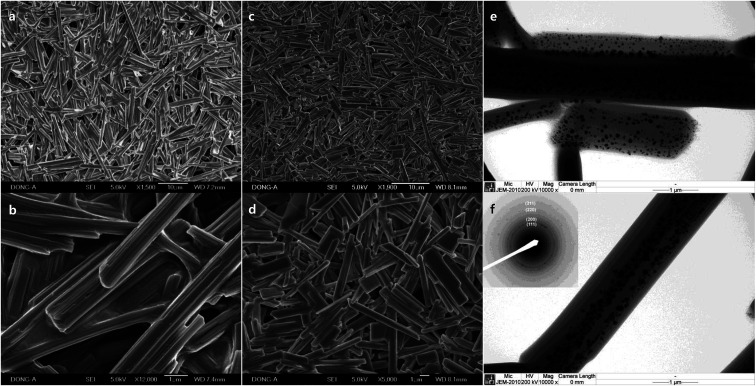
SEM images of different magnification of macroscale rods prepared with (a) and (b) Pluronic P-123 (17.9%), and (c) and (d) Pluronic F-108 (17.9%). (e) and (f) TEM images of the rods obtained with Pluronic P-123 (17.9%), and SAED pattern of Ag NPs in the inset of (f).

Macroscale rods were also obtained with Pluronic P-123 (17.9%, Fig. 4a and b), and Pluronic F-108 (17.9%, Fig. 4c and d). The high aspect ratio of P-123 (12.8) compared to L-64 (9.73) is due to the decrease of width, and not because of its length. It is notable that the average width of rods decreases with increasing *M*_n_ values. While the increase of PEO size does not alter significantly the size of micellar product, the increase of PPO blocks increases the size of micelles significantly.^34^ The chemical structures of Pluronic polymers in this study are EO_2_PO_16_EO_2_ (L-31), EO_13_PO_30_EO_13_ (L-64), EO_20_PO_69_EO_20_ (P-123), EO_265_PO_50_EO_265_ (F-108). Considering relatively high *M*_n_ of F-108 and the small dimension of rods, the low ratio of PO compared to EO (0.189) and the resulting small size of micelles are believed to affect structural characteristics. The high ratio of PO to EO in L-31 (8.0) contributes to the structural stability despite its low *M*_n_ value. Ag NPs with the average diameter of 46.9 nm (±18.0) were observed in the rods obtained with P-123 (Fig. 4e and f). Selective area electron diffraction pattern (SAED) indicates fcc structure of metallic silver (inset, Fig. 4f).

**Fig. 5:**
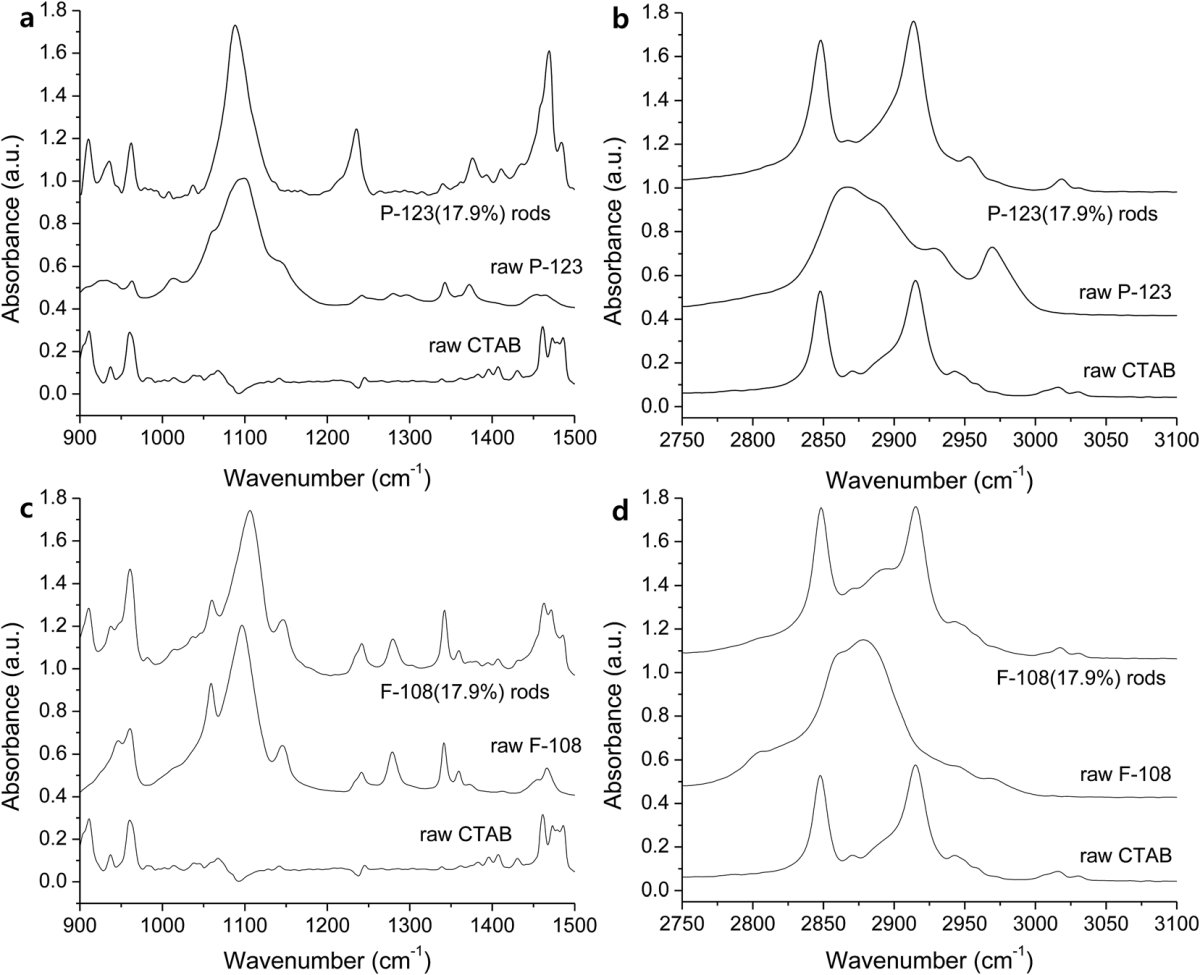
IR spectra of the rods prepared with Pluronic P-123 in the range of (a) 900–1500 cm^−1^ and (b) 2750–3100 cm^−1^, and with Pluronic F-108 in the range of (c) 900–1500 cm^−1^ and (d) 2750–3100 cm^−1^.

In FTIR spectra, rods obtained with P-123 (17.9%) show C–O stretching peak slightly red-shifted (Fig. 5a). The peak is also sharper (1090 cm^−1^, FWHM of 35.7 cm^−1^) compared to that of raw P-123 (1095 cm^−1^, FWHM of 59.8 cm^−1^). The peak at 1234 cm^−1^, which is absent in the raw CTAB and Pluronics, is observed in all the rods, in particular when more hydrophilic Pluronics (17.9%) are used. It is from CH_2_ twist modes from Pluronics. The intensity of CH_2_ wag at 1343 cm^−1^ and the symmetric deformation of CH_3_ at 1372 cm^−1^ increases due to the reduced hydrogen bonding and the self-assembly of dehydrated methyl groups with alkyl chains of CTAB. CTAB alkyls affect the shift of small peak at 2954 cm^−1^ (CH_3_ antisymmetric stretching, Fig. 5b). Similar to the rods obtained with L-64 (Fig. 3c), antisymmetric CH_3_ stretching (2970 cm^−1^) of Pluronics is completely oppressed. These results indicate that self-assembly in the hydrophobic region is driven by alkyls of CTAB, and the significant number of CH_2_ groups in CTAB dominates the spectra.

Contrary to the other rods, C–O stretching band at 1104 cm^−1^ is slightly blue-shifted compared to raw F-108 (1092 cm^−1^, Fig. 5c). However, the overall spectrum is similar to that of pure F-108. The structure of Pluronic F-108 is EO_265_PO_50_EO_265_. Ag(i) complexation within the hydrophilic EO regions does not affect the entire spectrum due to the large number of EO groups. Similarly, CH_2_ twist mode at 1235 cm^−1^, the antisymmetric CH_3_ stretching peak at 2980 cm^−1^, and CH_2_ wag at 1340 cm^−1^ are also believed to be pronounced due to the large number of EO and PO groups (Fig. 5c).

**Fig. 6:**
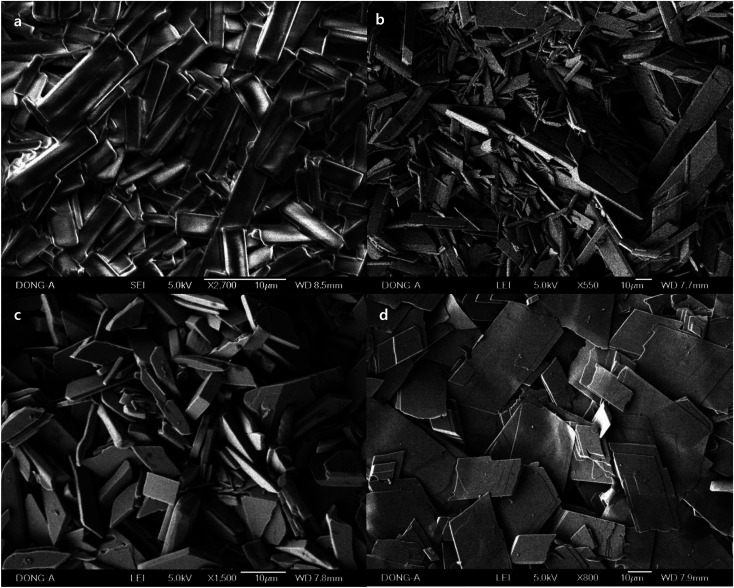
SEM images of the self-assembled rods prepared with Pluronics of (a) L-31 (17.9%), (b) L-64 (17.9%), (c) P-123 (17.9%), and (d) F-108 (17.9%) in the absence of ascorbic acid.

**Fig. 7:**
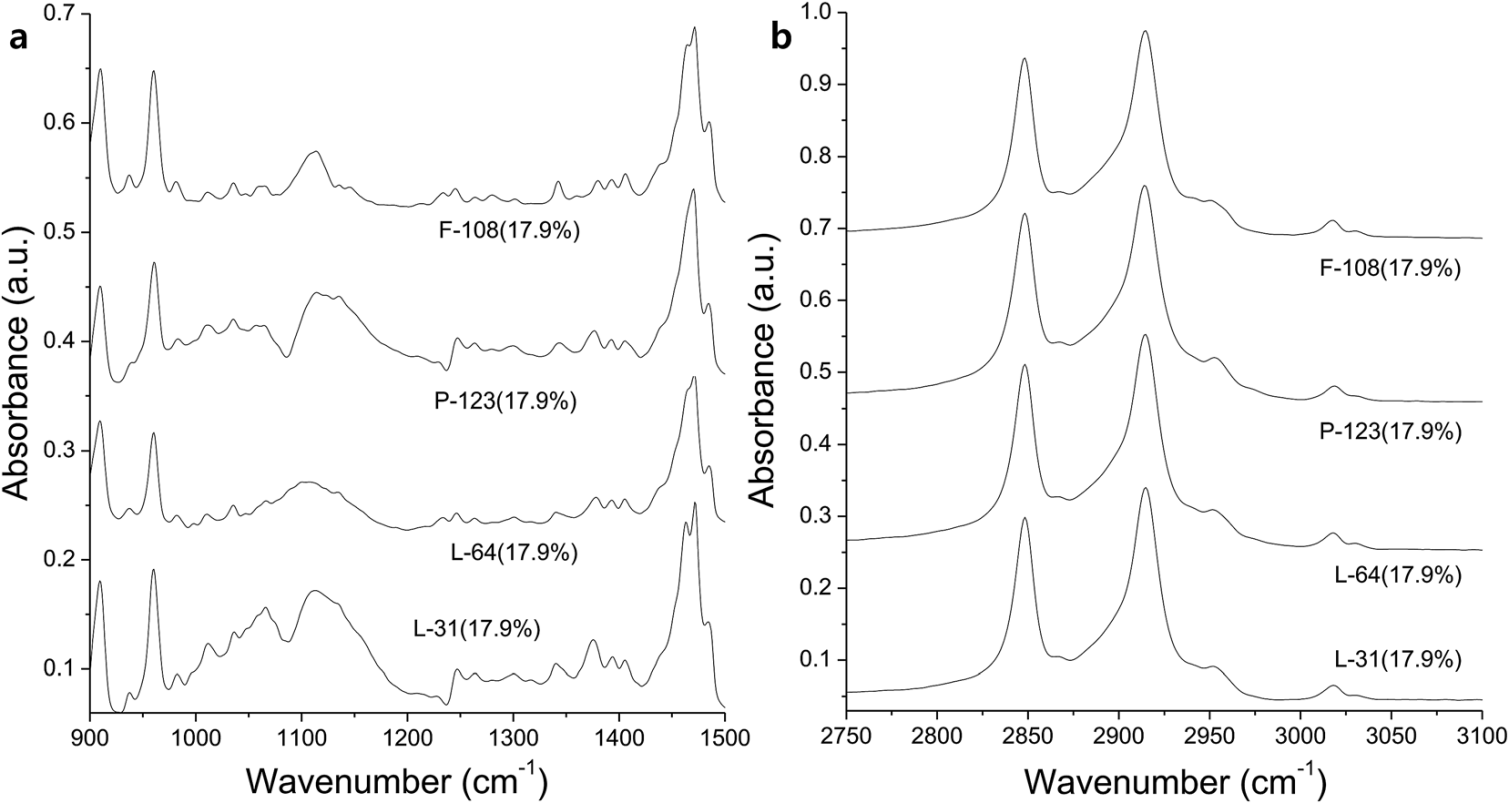
FTIR spectra of the products prepared with Pluronic copolymers in the absence of ascorbic acid, which are measured in the range of (a) 900–1500 cm^−1^ and (b) 2750–3100 cm^−1^.

When ascorbic acid is not used, the solution becomes more hydrophobic and CTAB is dominant in FTIR (Fig. 7). Rods were prepared with 17.9% concentration of Pluronics, which implies that the mixture is hydrophilic enough. However, C–O stretching band is weak and broad, and CH_2_ wag at 1340 cm^−1^ and CH_3_ deformation vibration around at 1370 cm^−1^ are not clearly observed (Fig. 7a). C–O stretching band moves to higher frequencies due to the increase of hydrophobicity and the spatial confinement (Fig. 7a). This hydrophobic environment is reflected on the broader and weaker C–O stretching, which is strikingly contrasted to those C–O stretching peaks that are obtained in the presence of ascorbic acid. The dimension of rods is micron scale, for example 26.0 μm × 17.9 μm (length × width) in case of F-108 (17.9%, Fig. 6d). It is believed that the major role for the production of macroscale materials is played by the self-assembly of CTAB and dehydrated methyl groups in PPO blocks. The charge density of CTAB, which is cationic surfactant, decreases in more hydrophobic environment. As it is known that the charged layer restricts self-assembly of hydrophilic headgroups of CTAB and Pluronics,^35^ self-assembly in the long range is enabled in more hydrophobic solution, thus producing larger rods and plates in the absence of ascorbic acid. The addition of ascorbic acid makes the mixture more hydrophilic at the elevated temperature, and is thought to hinder the self-assembly in the hydrophilic interface of corona-core region. AgNO_3_ in the absence of ascorbic acid is also to be considered. Generally, the addition of salt to Pluronics makes the solution more hydrophobic by the dehydration of water around the coronas, and therefore makes C–O stretching peak broader with the blue-shift of the peaks.^36^ This hydrophobic environment makes alkyl stretching bands dominant in FTIR. Dehydration by the increase of hydrophobicity in the absence of ascorbic acid is also reflected on the weak CH_2_ twist modes around at 1090 cm^−1^, which came from hydrophilic PEO coronas (Fig. 7a).

**Fig. 8:**
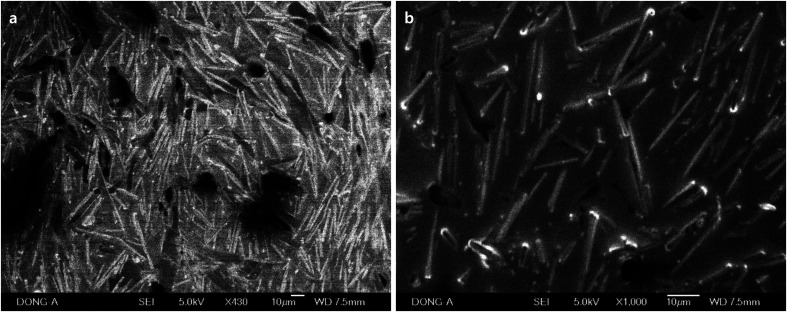
SEM images of self-assembled rods prepared with Pluronic P-123 (17.9%). They were prepared in the absence of AgNO_3_ and ascorbic acid.

In order to study the effect of silver nitrate on the growth of 1D rods, the experiments in the absence of AgNO_3_ and ascorbic acid were conducted. Rods from Pluronic copolymers (17.9% aqueous P-123) were observed, but they are imbedded in CTAB solid matrix and do not exist as separable solids (Fig. 8). Rods were only observed in P-123 copolymers in the absence of AgNO_3_. This might be due to the intrinsic stability of the self-assembled materials when P-123 is used.

**Fig. 9:**
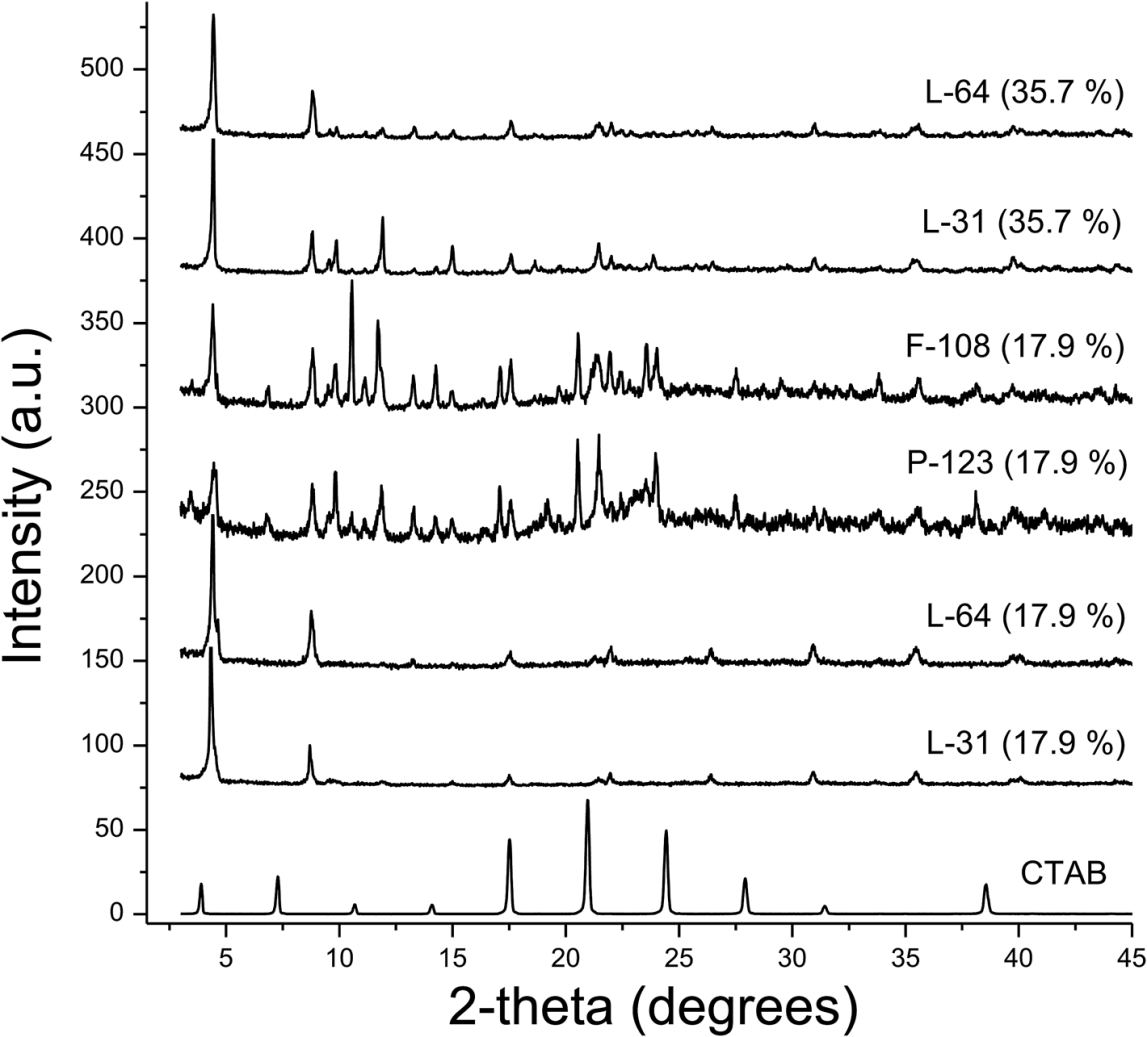
XRD patterns of macroscale rods that are obtained with 17.9% and 35.7% aqueous solution of Pluronics. The pattern of crystallized CTAB is added at the bottom of the figure.

XRD patterns of these macroscale rods indicate that the lamellar structures are prevalent (Fig. 9). The crystallized CTAB maintains layer-by-layer structure with a *d*-spacing of 22.6 Å (2-theta = 3.90°, bottom of Fig. 9). In rods and plates, however, the peaks of CTAB are hardly observed, though similar lamellar packing with slightly smaller *d*-spacings ranging from 19.9 Å (L-64, 17.9%) to 20.4 Å (L-64, 35.7%) is identified from the first (*n* = 1) peaks. With the addition of AgNO_3_, CTAB crystallization is inhibited. The addition of salts to Pluronics favors the anisotropic growth of micelles. With the increase of hydrophobicity and consequently the decrease of critical micelle temperature, Pluronics with AgNO_3_ are believed to produce anisotropic micelles at lower temperatures. Rheology and rigidity as well as the morphology of nanofibers generated from the silver(i)-coordinated metallogels are also dependent on the types of silver anions.^37^ In fact, Ag(i) has been widely used for constructing 1D soft materials, as Ag(i) is a flexible metal cation which binds diverse heteroatoms. Its ability to coordinate linearly with a coordination number of two enables the formation of flexible 1D materials.^38,39^ AgNO_3_ in this study is critical to obtain 1D and 2D soft solids. With the effect of salt on the tendency to be crystalline structure, and during the annealing at 70 °C, all Pluronics are thought to form crystalline mesophases. Salt resides in the PEO coronas, breaking hydrogen bonding between water and EO groups. Dehydration of PEO coronas either by the coordination of transition metal cation or by the effect of counter anions causes the increase of hydrophobicity.

**Scheme 1:**
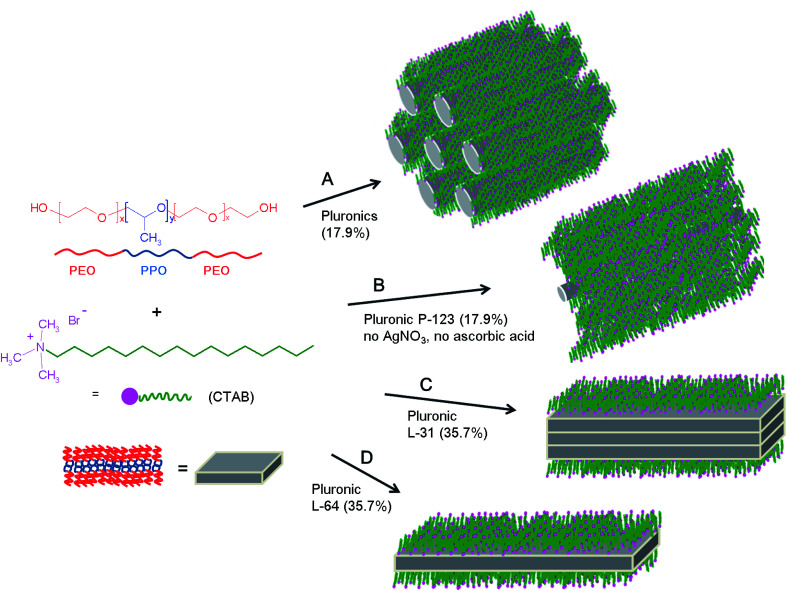
The process for the formation of macroscale rods and plates from the surfactant mixture of CTAB and Pluronics in the presence (A, C and D) and in the absence (B) of AgNO_3_ and ascorbic acid.

The plausible formation mechanism of macroscale rods and plates is illustrated in Scheme 1. Pluronics in general prefer anisotropic micelles at the elevated temperature. When the temperature increases, PEO coronas near hydrophobic PPO groups are dehydrated and they are combined with hydrophobic cores, thus hydrophobic micellar cores become larger. In order to minimize the shape instability of the spheres, the spherical micelles change to ellipsoidal shape, and further to cylindrical or rod-like micelles in hexagonal geometry when the temperature is increased (mechanism A). In these micellar cores, Ag(i) ions are believed to be capped and they also play roles to bridge micelles nearby. The addition of inorganic salt in fact favors the formation of cylindrical micelles at lower temperature.^40^ Without the addition of AgNO_3_, small cylindrical micelles are imbedded in CTAB matrix, and the formation of these cylindrical micelles is simply contributed by the self-assembly of alkyl chains of CTAB and the methyl groups of PPO blocks in Pluronic P-123 (mechanism B). The chemical formula of Pluronic P-123 is EO_20_PO_69_EO_20_, and it is regarded as the most hydrophobic Pluronic copolymers. This high hydrophobicity is assumed the reason that rods are only observed with Pluronic P-123 in the absence of AgNO_3_ and ascorbic acid. When the concentration of Pluronic copolymers is increased to 35.7%, lamellar plates are observed. Layer-by-layer anisotropic lamellar mesophases are normally observed when the concentration of Pluronics is highest.^10^ The small hydrophilic corona areas, which are formed by the small number of EO groups in EO_2_PO_16_EO_2_ (Pluronic L-31), are not able to separate hydrophobic lamellar plates, and the stacked plates are produced (mechanism C). In Pluronic L-64 (EO_13_PO_30_EO_13_), however, relatively large number of EO groups can efficiently separate each plate through hydrophilic coronas (mechanism D). These hydrophilic areas are composed of the ammonium head group of CTAB and PEO groups in which chains of linear O–Ag–O bonding occurs. Lamellar formation of silver carboxylates are well known,^28,41^ and based on this layer-by-layer formation of micronscale rods are obtained with silver carboxylates.^42^

## Conclusion

Anisotropic soft solids grown into micron scale 1D rods and 2D plates are produced without adding co-solvents which are typically used for solubilizing selective blocks. Rigidity is afforded by the addition of silver nitrate to the mixture of Pluronic copolymers and CTAB, and the aspect ratio is dependent on the number average molecular weight and the hydrophobicity of block copolymers. In the absence of ascorbic acid, similar but much larger rods and plates are produced. The effects of ascorbic acid are (i) the increase of hydrophilicity and the better complexation of Ag(i) within the EO moiety, (ii) the disruption of the long range self-assembly in the hydrophilic interface of core–corona. Through this hindrance, smaller size of rods and plates are produced in the presence of ascorbic acid. XRD patterns indicate that layer by layer stacking is the main theme for constructing anisotropic rods and plates. This lamellar packing is thought the collaboration of CTAB, Pluronics and Ag(i), as Pluronics with the highest concentration favor the formation of lamellar mesophases at the elevated temperature and many silver carboxylates containing long alkyl chains as the side group produce metallogels as micronscale one-dimensional materials.

## Conflicts of interest

The author declares no competing financial interest.

## Supplementary Material

RA-009-C8RA10517K-s001
