# Supplementary material for: Ag(i)-mediated self-assembly of anisotropic rods and plates in the surfactant mixture of CTAB and Pluronics
Source: RSC Adv. 2019 Feb 5;9(8):4380–9. doi: 10.1039/c8ra10517k (PMC9060553; doi:10.1039/c8ra10517k)
Supplement: RA-009-C8RA10517K-s001 [file RA-009-C8RA10517K-s001.pdf]

Electronic Supplementary Material (ESI) for RSC Advances.
This journal is © The Royal Society of Chemistry 2019

Electronic Supplementary Information (ESI)

Ag(I)-Mediated Self-Assembly of Anisotropic Rods and Plates in the Surfactant Mixture of CTAB and Pluronics

Hyon-Min Song[†,*] *and Jeffrey I. Zink*[‡]

[†]Department of Chemistry, Dong-A University, Busan 604-714, South Korea

[‡]Department of Chemistry and Biochemistry, University of California, Los Angeles, Los Angeles, California 90095-1569, USA

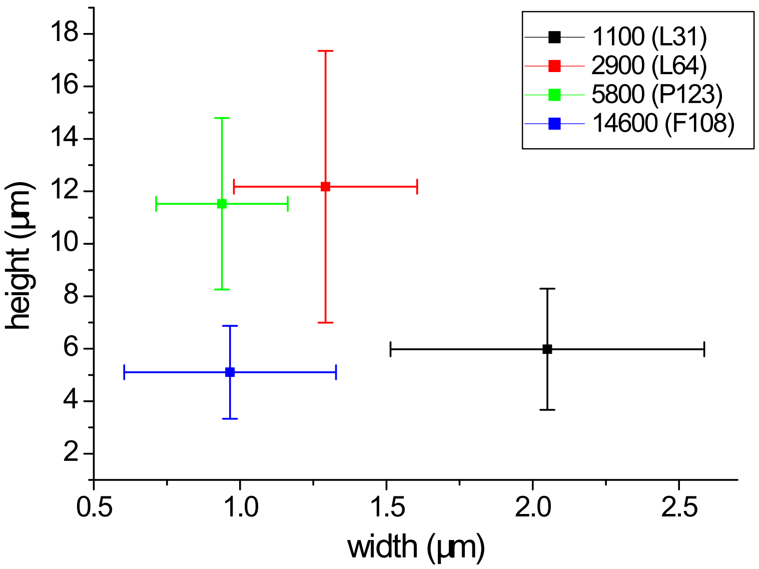

Fig. S1 Dimension with the error bars of the rods that are prepared with Pluronics (17.9%)

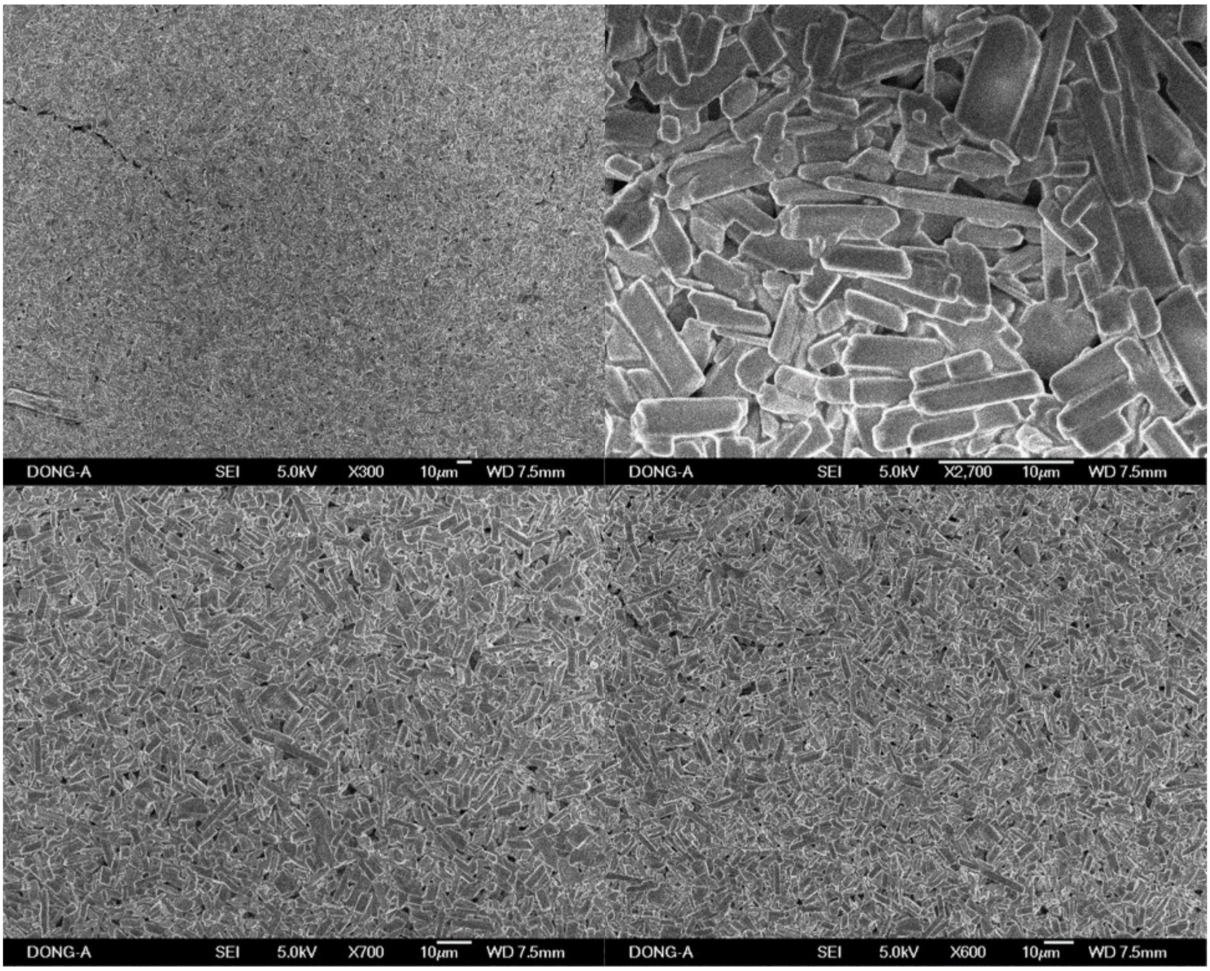

Fig. S2 SEM images of different magnification of the self-assembled rods prepared with Pluronic L-31 copolymers (17.9% aqueous, Mn~1,100).

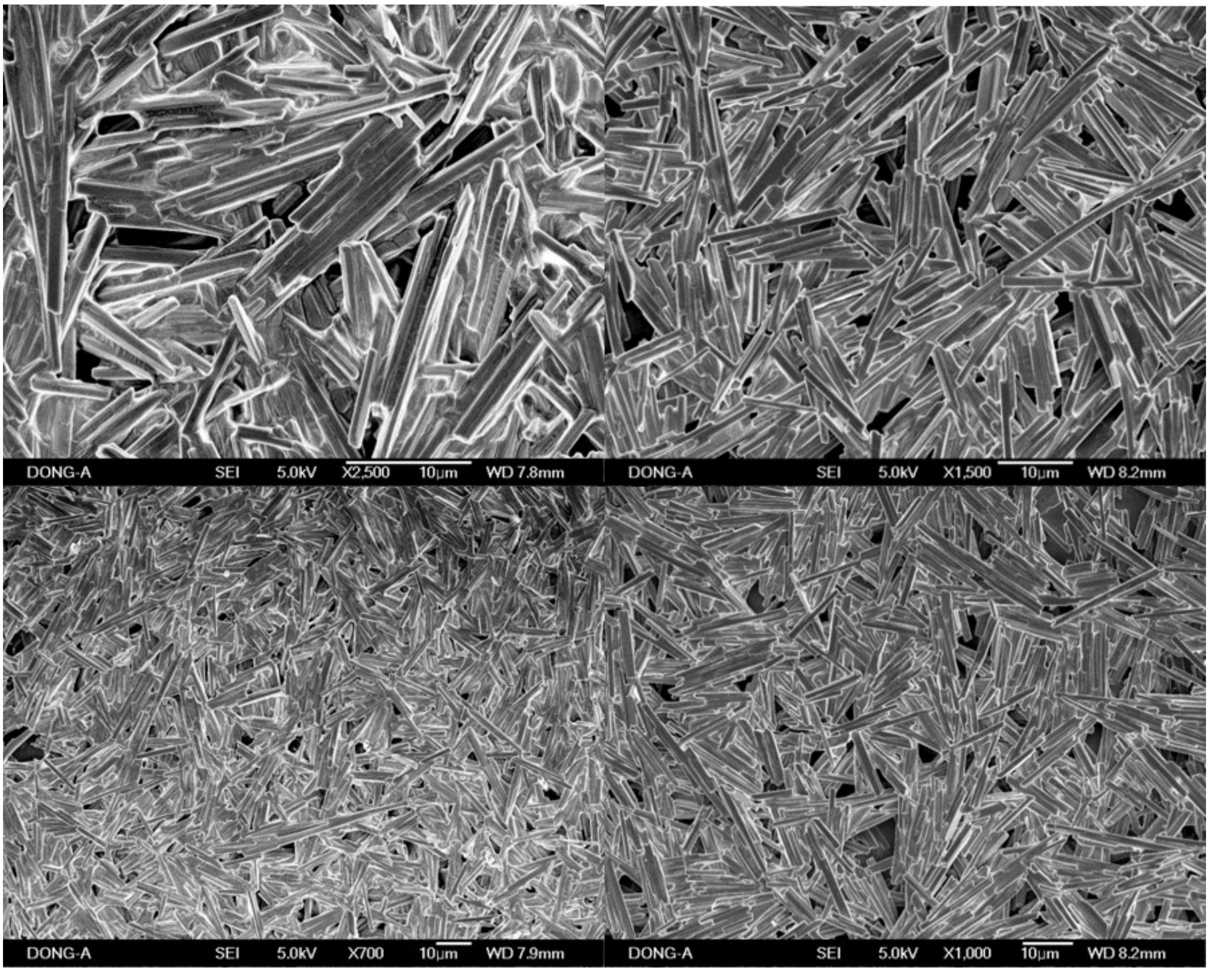

Fig. S3 SEM images of different magnification of the self-assembled rods prepared with Pluronic L-64 copolymers (17.9% aqueous, Mn~2,900).

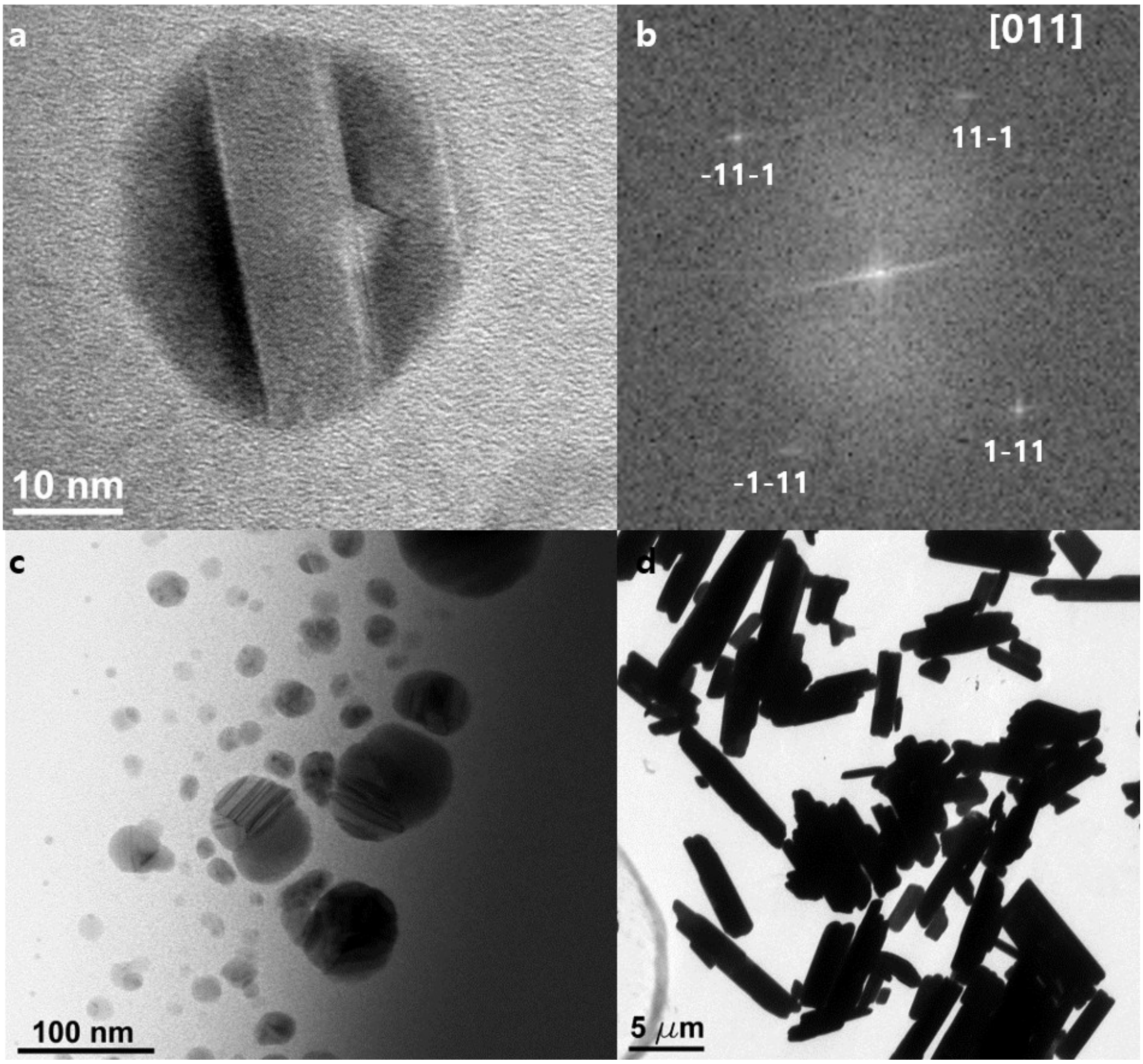

Fig. S4 (a), (c) TEM images of nanoparticles imbedded in the rods prepared with Pluronic L-64 copolymers (17.9% aqueous, Mn~2,900). (b) SAED pattern of the TEM image in (a). (d) TEM image of the self-assembled rods.

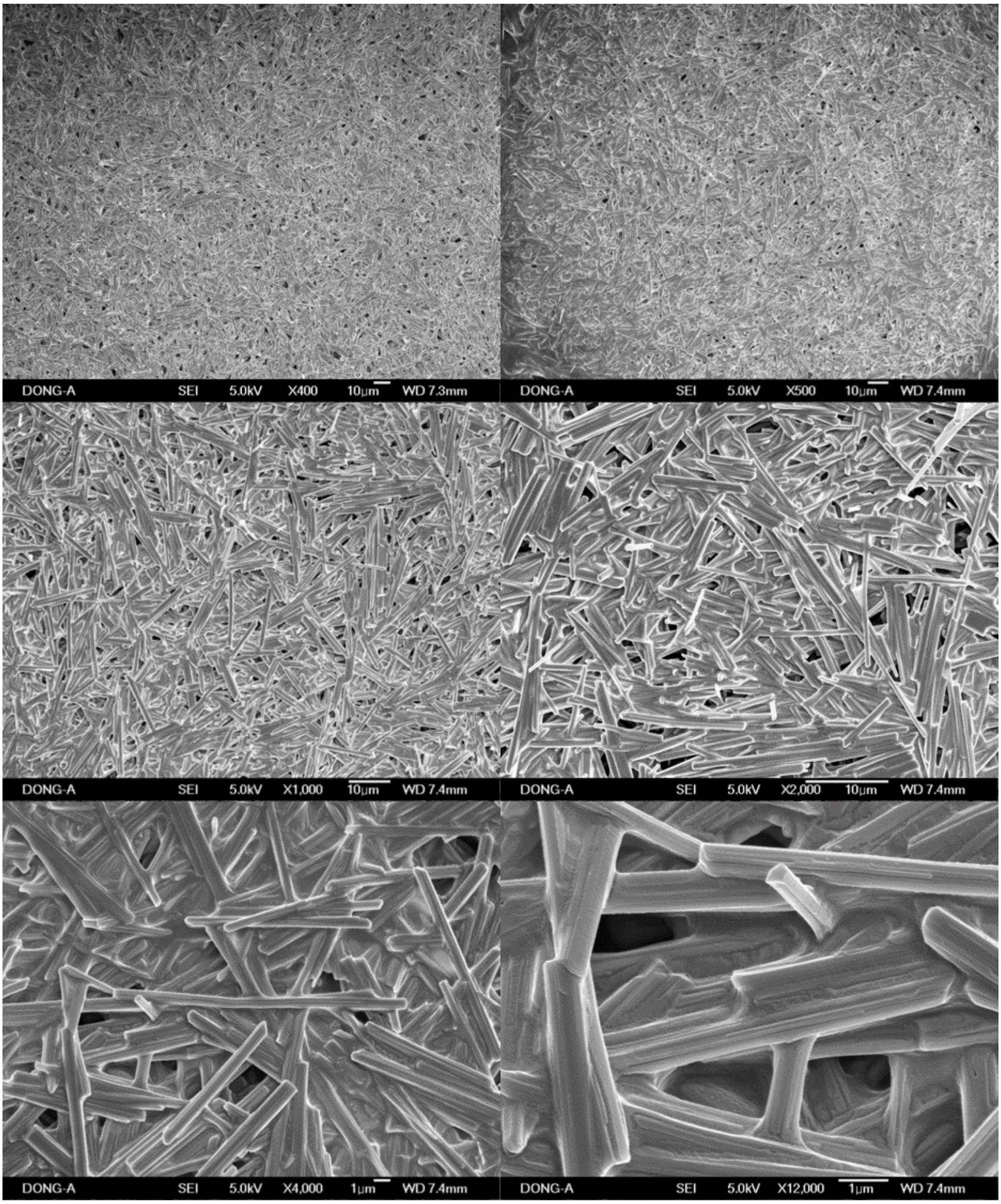

Fig. S5 SEM images of different magnification of the self-assembled rods prepared with Pluronic P-123 copolymers (17.9% aqueous, Mn~5,800).

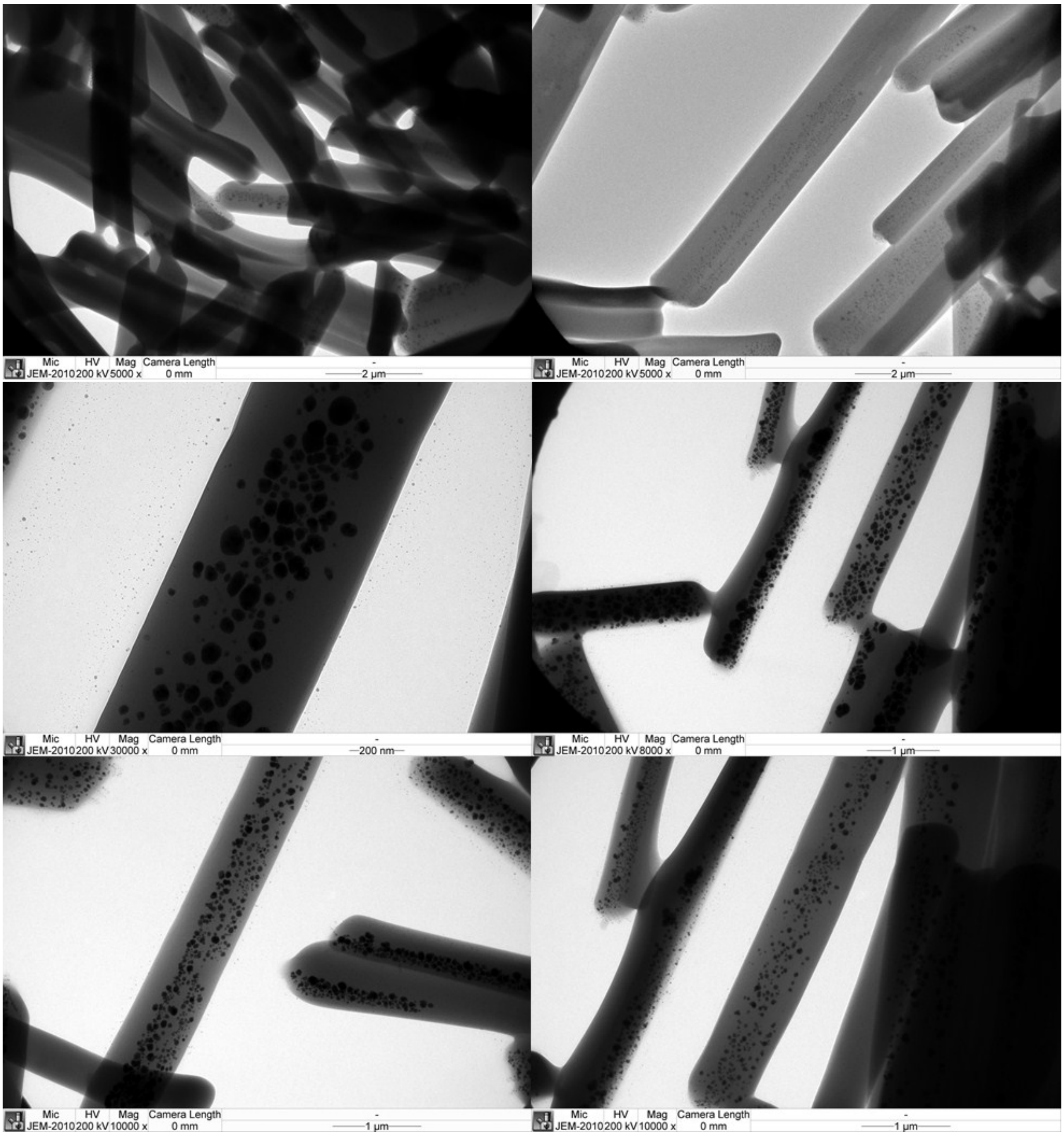

Fig. S6 TEM images of different magnification of the self-assembled rods prepared with Pluronic P-123 copolymers (17.9% aqueous, Mn~5,800).

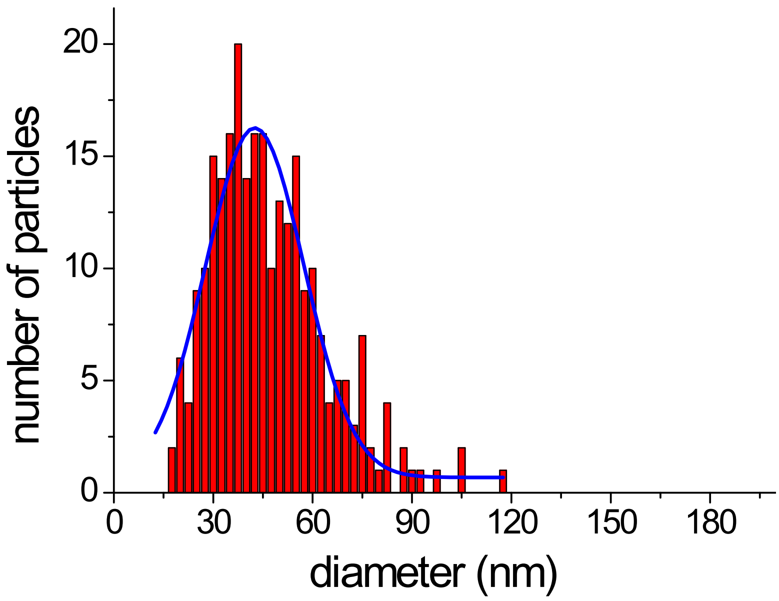

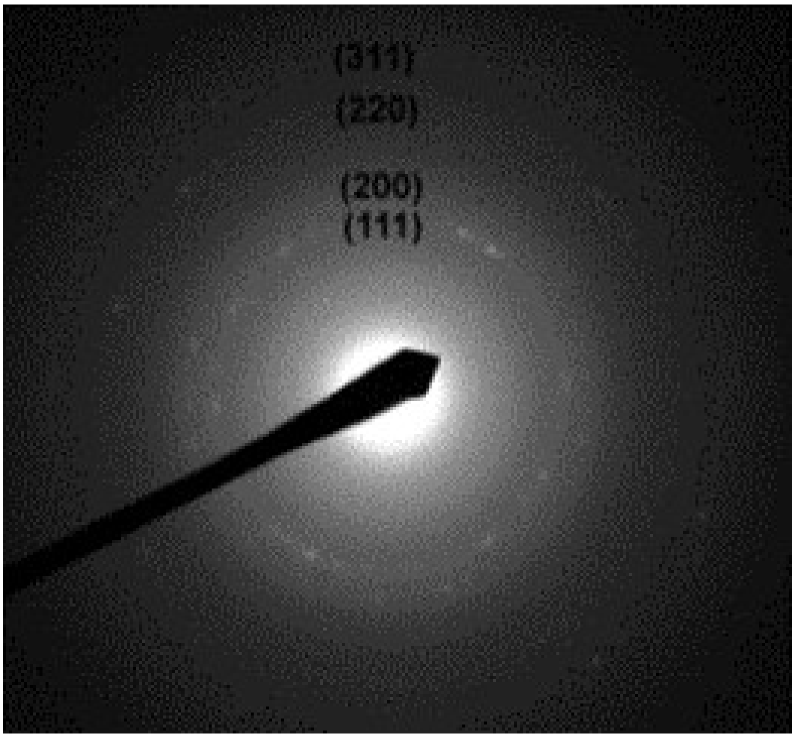

Fig. S7 (top) Size distribution and (bottom) SAED pattern of nanoparticles that are imbedded in the rods. The rods were prepared with Pluronic P-123 copolymers (17.9% aqueous, Mn~5,800).

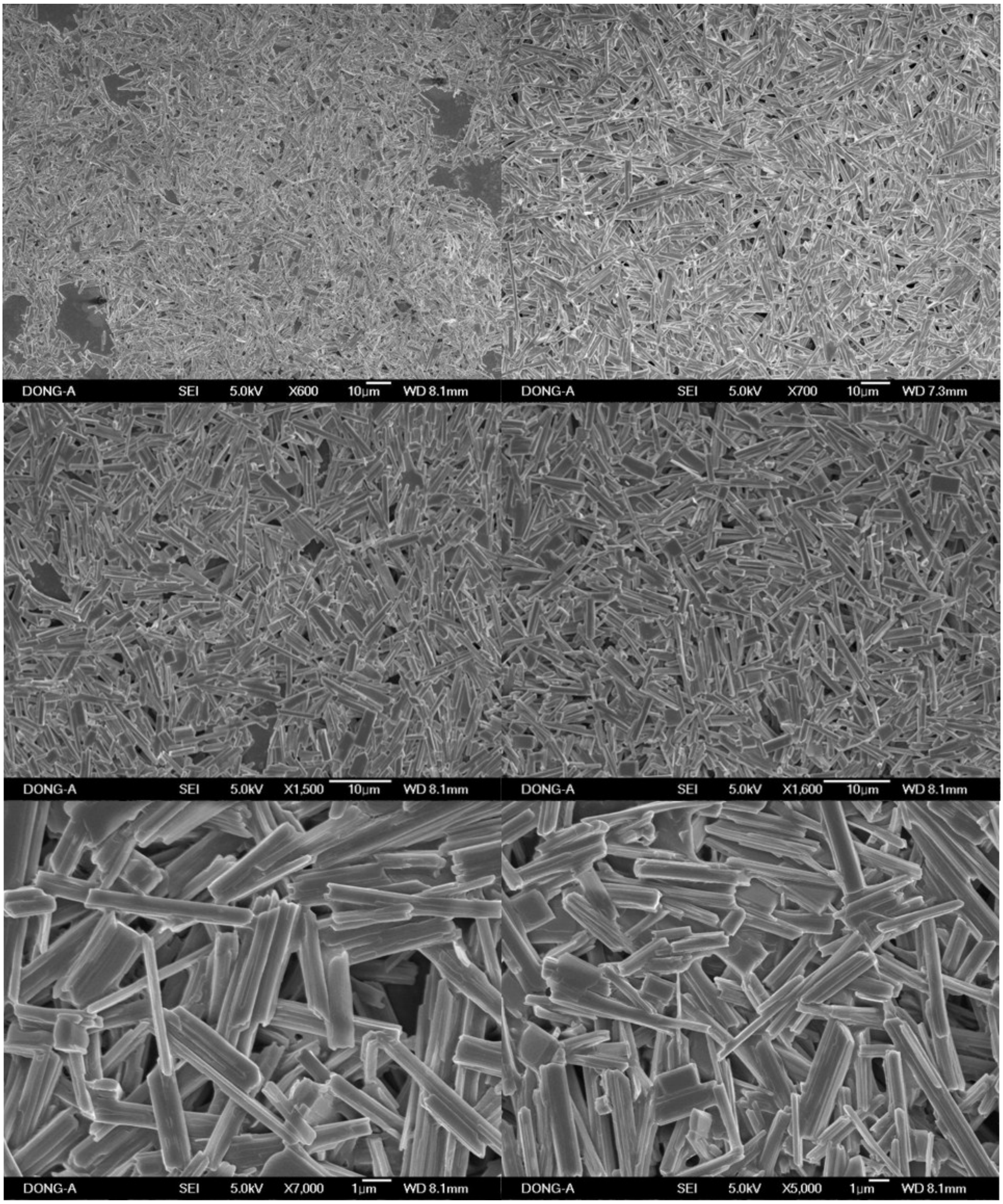

Fig. S8 SEM images of different magnification of the self-assembled rods prepared with Pluronic F-108 copolymers (17.9% aqueous, Mn~14,600).

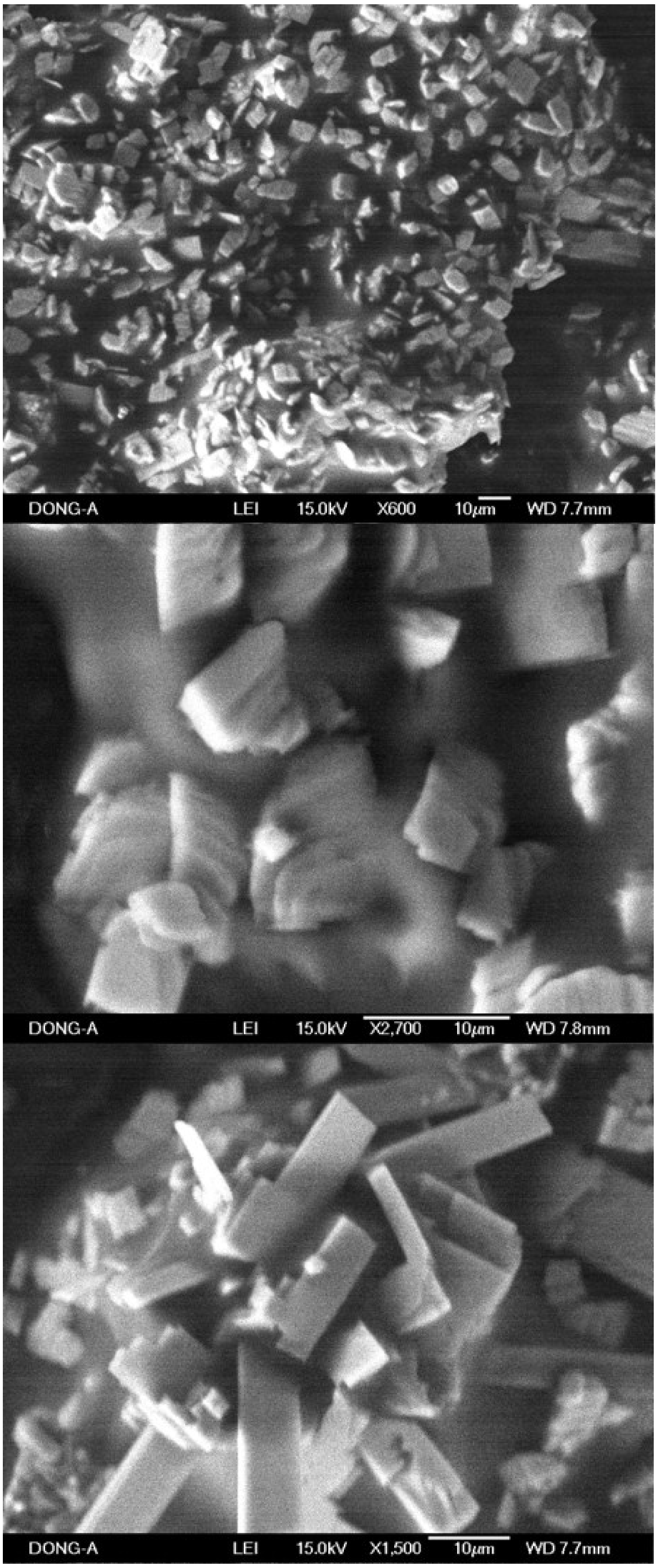

Fig. S9 SEM images of different magnification of the self-assembled rods prepared with Pluronic L-31 copolymers (35.7% aqueous, Mn~1,100).

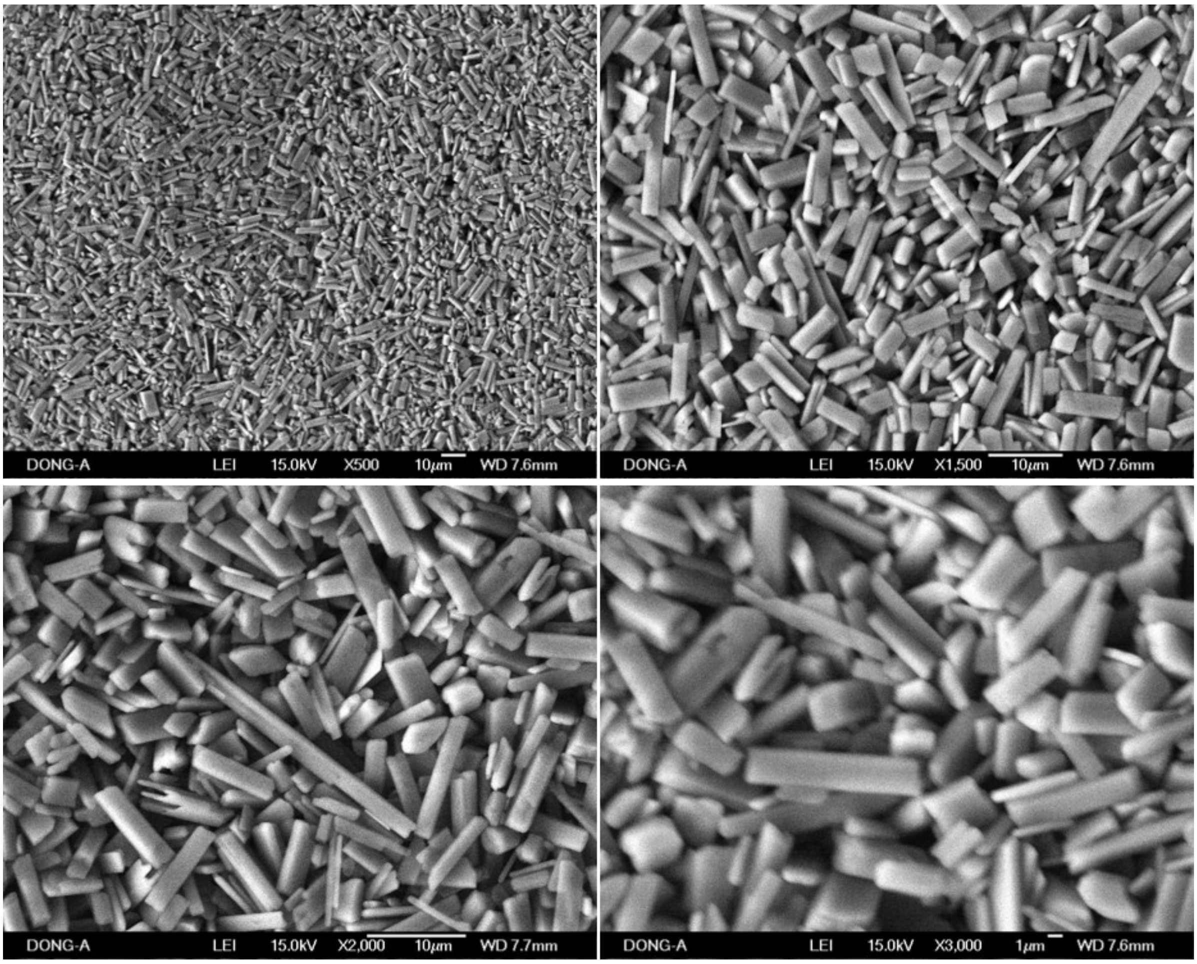

Fig. S10 SEM images of different magnification of the self-assembled rods prepared with Pluronic L-64 copolymers (35.7% aqueous, Mn~2,900).

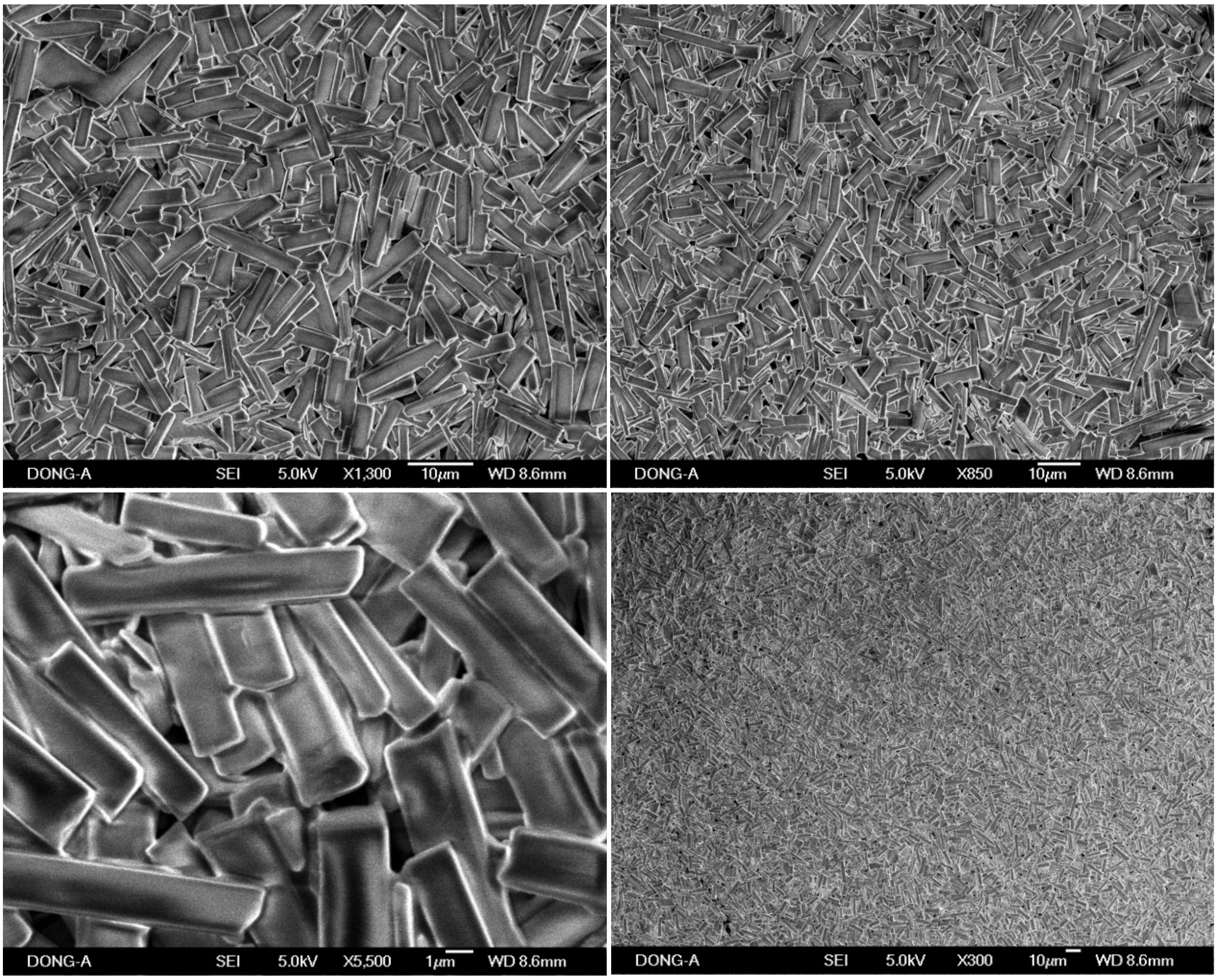

Fig. S11 SEM images of different magnification of the self-assembled rods prepared with Pluronic L-64 copolymers (17.9% aqueous, Mn~1,100) in the absence of ascorbic acid.

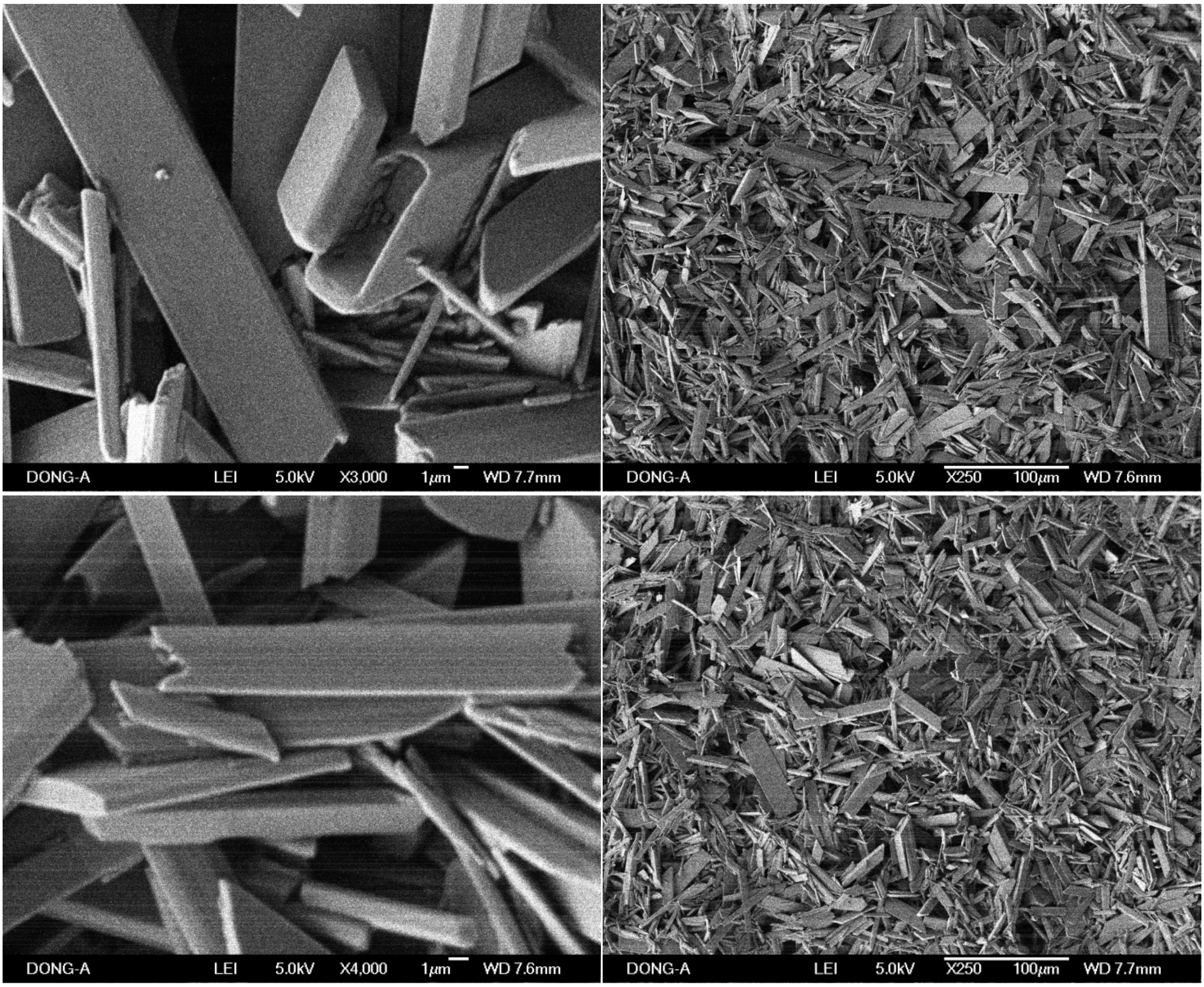

Fig. S12 SEM images of different magnification of the self-assembled rods prepared with Pluronic L-64 copolymers (17.9% aqueous, Mn~2,900) in the absence of ascorbic acid.

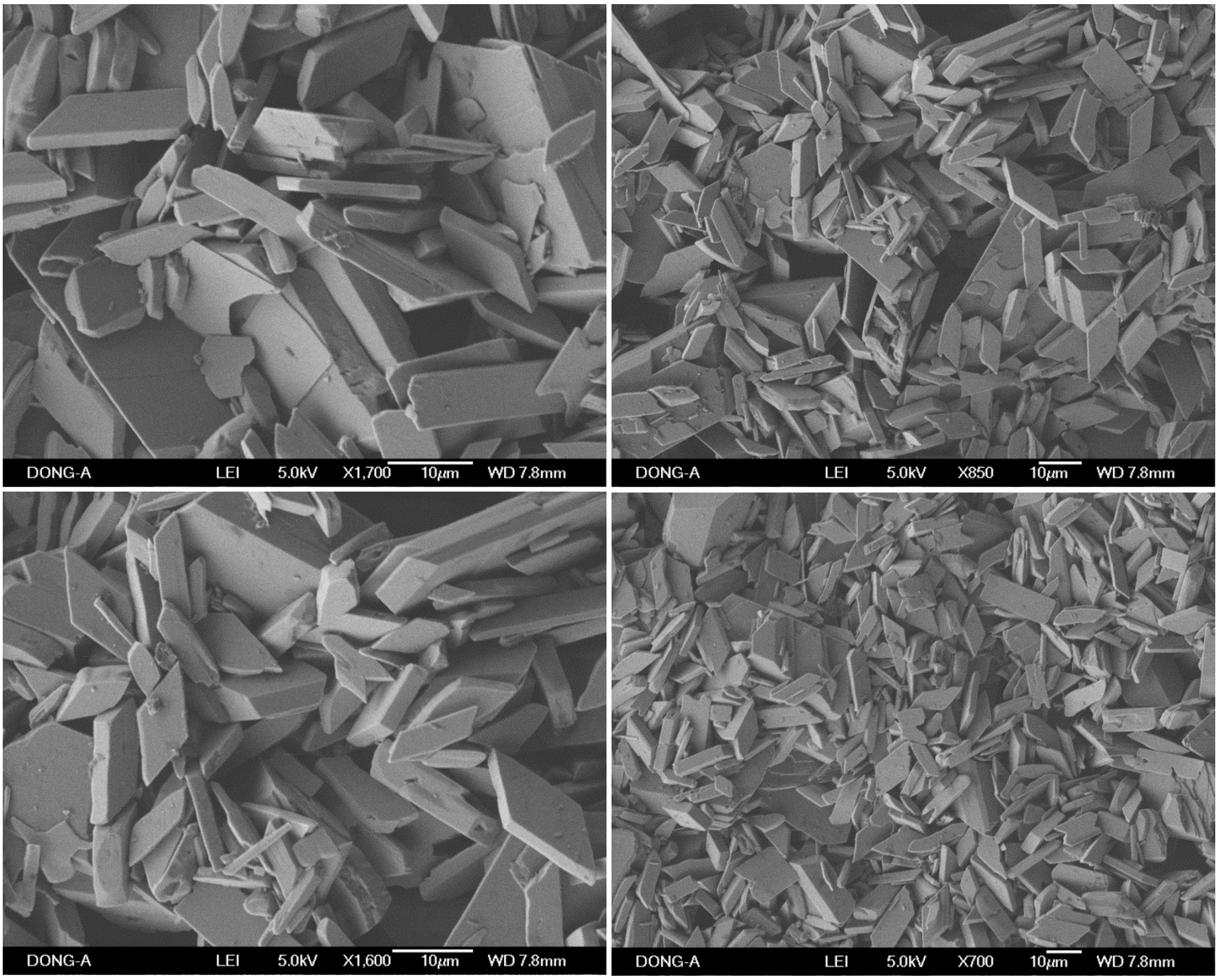

Fig. S13 SEM images of different magnification of the self-assembled rods prepared with Pluronic P-123 copolymers (17.9% aqueous, Mn~5,800) in the absence of ascorbic acid.

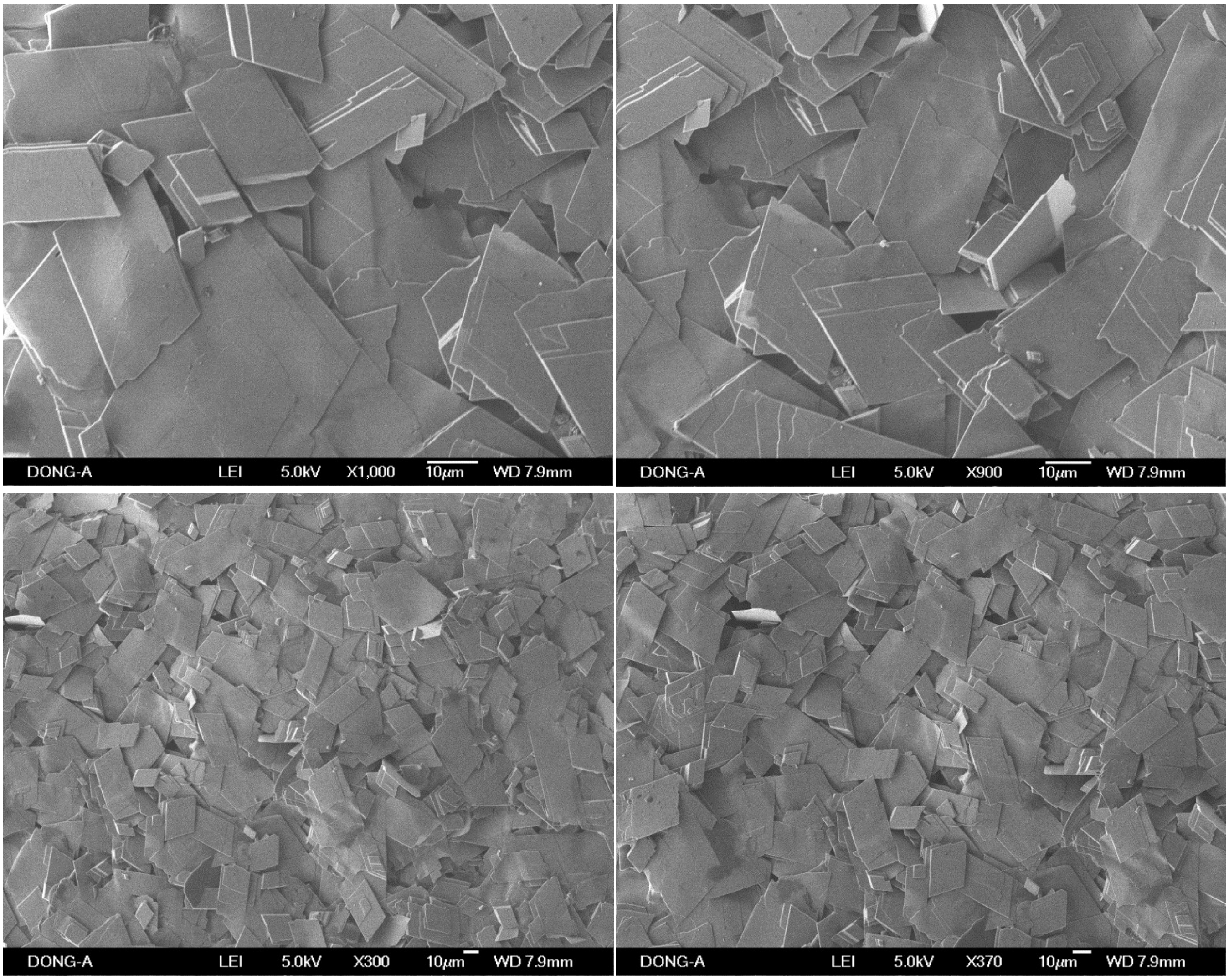

Fig. S14 SEM images of different magnification of the self-assembled rods prepared with Pluronic F-108 copolymers (17.9% aqueous, Mn~14,600) in the absence of ascorbic acid.

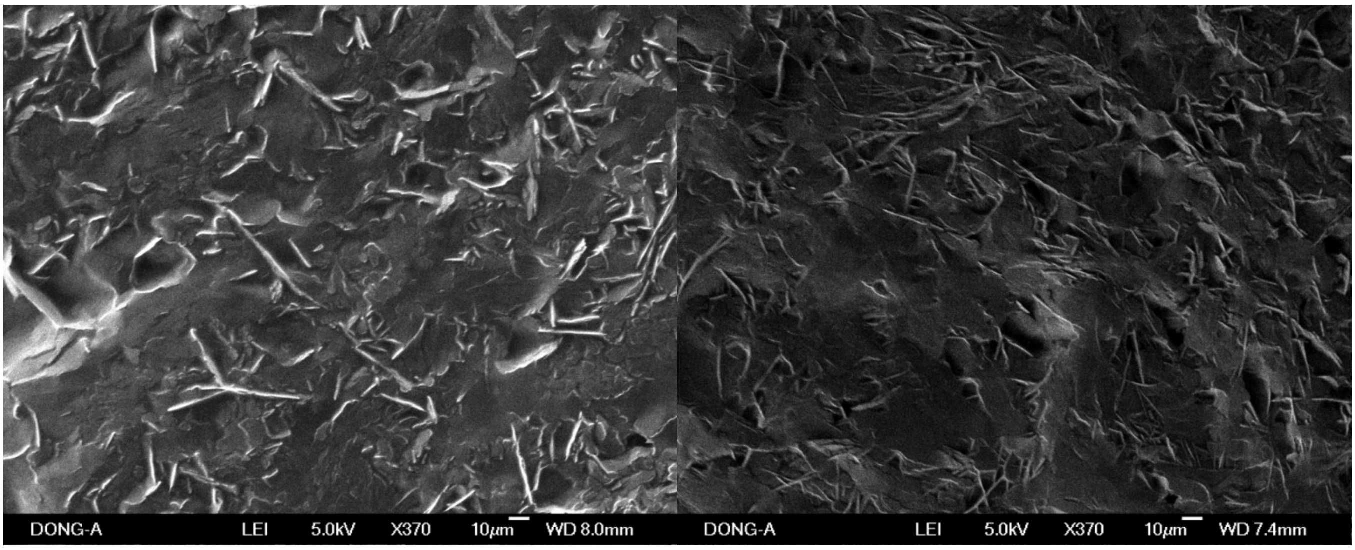

Fig. S15 SEM images of the products which were obtained in the absence of Pluronic copolymers.

Table S1. Calculated *d*-spacings of the first and second peaks in the XRD patterns of self-assembled rods

sample	First peak (degrees)	*d*-spacing	Second peak (degrees)	*d*-spacing
CTAB	3.90	22.6 Å	7.28	12.1 Å
L-31 (17.9%)	4.43	19.9 Å	8.82	10.0 Å
L-64 (17.9%)	4.43	19.9 Å	8.80	10.0 Å
L-31 (35.7%)	4.41	20.0 Å	8.78	10.1 Å
L-64 (35.7%)	4.33	20.4 Å	8.71	10.1 Å
